# Trends of incident adult Attention-deficit/hyperactivity disorder diagnoses before, during and after the pandemic provincial state of emergency in British Columbia (2013–2023): a population-based study

**DOI:** 10.1016/j.lana.2025.101223

**Published:** 2025-09-16

**Authors:** Kevin Hu, Roshni Desai, Shania Au, Bin Zhao, Skye Barbic, Kirsten Marchand, Tonia Nicholls, Christian Schütz, Hasina Samji, Jia Hu, Geoff McKee, Alexis Crabtree, Heather Palis

**Affiliations:** a BC Centre for Disease Control, Vancouver, Canada; b School of Population and Public Health, University of British Columbia, Vancouver, Canada; c Department of Occupational Science and Occupational Therapy, University of British Columbia, Vancouver, Canada; d Department of Psychiatry, University of British Columbia, Vancouver, Canada; e Faculty of Health Sciences, Simon Fraser University, Burnaby, Canada; f Foundry, 1045 Howe St #915, Vancouver, Canada

**Keywords:** Substance use, ADHD, Mental health, Pandemic

## Abstract

**Background:**

Emerging evidence indicates a potential rise in attention-deficit/hyperactivity disorder (ADHD) incidence worldwide and in British Columbia (BC) since the pandemic. Given the high comorbidity of ADHD with substance use disorder (SUD) and other mental disorders, understanding changes in ADHD diagnosis among adults is crucial for healthcare planning amid BC's drug poisoning (overdose) crisis. We aimed to report how rates of newly diagnosed adult ADHD changed before, during and after the pandemic by demographic variables and histories of SUD or mental disorders.

**Methods:**

We conducted interrupted time series analyses on overall and stratified monthly incidence rates of ADHD diagnosis between Jan, 2013, and Nov, 2023 in BC, using data from linked population-based administrative databases.

**Findings:**

The pre-pandemic average of newly diagnosed adult ADHD was 8.8 cases per 100,000 population monthly. During the pandemic (Mar, 2020–Jun, 2021), this rose to 19.2 driven by a 4.9% (95% confidence interval: [3.7, 6.2]) month-over-month increase. When the pandemic ended, the monthly rate jumped by 107.3% [68.5, 155.0] in Jul, 2021 and grew 1.5% [0.4, 2.7] per month thereafter, averaging 34.8 cases per 100,000 post-pandemic. Substantial differences in trends emerged when stratified by sex and SUD histories.

**Interpretation:**

This exponential rise in adult ADHD may be explained by pandemic-related sociocultural changes and the broader societal evolution in mental health awareness in recent years and decades. This rise could foreshadow a potential increase in the population at risk of SUD, underscoring the urgent need for bidirectional integration of ADHD and SUD services.

**Funding:**

We acknowledge the UBC Psychiatry Stimulus Grants Initiative.

**Research in context**

**Evidence before this study**

Attention-deficit/hyperactivity disorder (ADHD) is a well-established risk factor for substance use disorders (SUD) and mental health conditions. Understanding changes in ADHD diagnosis patterns among adults is crucial for healthcare planning, particularly in the context of rising public awareness of ADHD and the ongoing drug poisoning crisis in North America. Emerging evidence indicates a rise in adult ADHD incidence worldwide in recent years. We searched Ovid MEDLINE for studies examining adult ADHD incidence using the search strategy “((newly diagnosed or new or inciden∗) adj3 (ADHD or Attention Deficit or Attention-Deficit)).ti,ab,kf.) AND Adult/” limited to publications after 2020. Our search yielded 57 results, of which 10 were relevant. Most of these studies used pre-pandemic data, reporting more than 2-fold increases in adult ADHD diagnoses and medication prescriptions in six European and Asian countries over the past two decades. Only one study, a Finnish national cohort study (2015–2022) specifically examined pandemic-related changes, finding that new ADHD diagnoses doubled during the pandemic, with particularly dramatic increases among females. We found no research on adult ADHD incidence rates in North America during the pandemic, with one Canadian study ending in 2015.

**Added value of this study**

Our study provides the first comprehensive examination of adult-specific ADHD incidence trends before, during, and after the COVID-19 pandemic in British Columbia, Canada. In addition to stratify by demographic variables, we examined changes in rates of newly diagnosed adult ADHD by histories of substance use disorders or mental disorders. We identified a nearly four-fold overall increase in incidence ADHD cases since the pandemic in British Columbia, with rates growing faster in females and eventually surpassing those in males. This aligns with international trends but exceeds the magnitude of increases reported in other jurisdictions. We also observed that rates grew slower in people with a SUD history than in people without SUD history, an unexpected finding given the high comorbidity of ADHD and SUD.

**Implications of all the available evidence**

The combined evidence indicates a substantial global increase in adult ADHD diagnoses, accelerated during the pandemic period. This substantial rise may reflect improved recognition of previously underdiagnosed ADHD, particularly in females, but also represents a growing population at future risk for developing SUD and experiencing drug poisoning events. The observed slower growth in rates among individuals with a SUD history in our study may reflect barriers that this population faces when accessing mental health and substance use care. Healthcare systems should prepare for increased demand for adult ADHD assessment and treatment services with integration of substance use services to minimize potential negative impacts while ensuring equitable access.

## Introduction

Attention-deficit/hyperactivity disorder (ADHD) is a neuro-developmental psychiatric disorder defined by impaired levels of attention and hyperactivity-impulsivity.^1^ Adult ADHD prevalence estimates range between 2.5 and 2.9%^2^ in Canada, similar to global estimates of the disorder in adults.^3^

In recent years, there has been growing attention to ADHD across the life course and increasing rates of incident diagnoses of ADHD internationally. For example, a recent population-based study in Finland found a doubling of new ADHD diagnoses between 2020 and 2022, and a 3-fold increase in females aged 13–30.^4^ While trends were showing a rising incidence of ADHD in many regions before the pandemic,^5^ the acceleration of ADHD diagnoses since 2020 has been attributed to consequences of the pandemic on mental health, improved and expanded education around ADHD, and possible changes in diagnostic practice since the pandemic, such as telemedicine.^6^

In British Columbia (BC), a previous study found a potential rise in mental health-related service visits and psychotropic drug dispensations among adults between 2019 and 2021.^7^ While these data indicate potential increases in treatment, to our knowledge, incidence rates of ADHD in adults have not been examined in BC, particularly important is to attend to key periods, such as pre- and post-pandemic provincial state of emergency. Furthermore, the profile of people with new diagnoses remains unknown, particularly with respect to history of comorbid mental and substance use disorder diagnoses.

Psychiatric comorbidity of ADHD with substance use disorders (SUDs) and other mental disorders is common. Prior studies have identified significant differences in prevalence and incidence rates of ADHD among people with mental health and/or substance use disorders.^8^ For example, estimates of ADHD prevalence in people with SUDs are up to ten times higher than in the general population.^9^ While estimates range across various mental disorder diagnoses, the World Mental Health Surveys report that nearly 20% of adults with ADHD also have diagnoses of three or more other classes of mental disorders.^10^ Overlapping diagnostic criteria between ADHD, SUDs, and other mental disorders may contribute to under or misdiagnoses of ADHD.^11^

Given the recognized historical overrepresentation of SUD and mental disorder diagnoses among people with ADHD, and the known impacts of the pandemic on mental health and well-being across the population of BC,^7^ this study has two objectives: 1) to report rates of adult ADHD incidence in BC over time (2013–23), across pre-pandemic, pandemic, and post-pandemic periods; and 2) to report on differences in trends of adult ADHD incidence by demographic variables and by mental or substance use disorder history.

## Methods

### Study design

We analyzed the changes in diagnosed ADHD incidence among adults in BC between Jan 1, 2013, and Nov 30, 2023, focusing on the impact of the COVID-19 pandemic. The study period was divided into three segments: before, during, and after the pandemic provincial state of emergency in BC.^12^ The provincial state of emergency was started on Mar 17, 2020, and lifted on Jun 30, 2021, repealing several orders and restrictions on gatherings and the requirement of face coverings in indoor public spaces. These periods are herein referred to as pre-pandemic, pandemic, and post-pandemic periods. Changes in diagnostic incidence of ADHD across periods were quantified and analyzed using interrupted time series analysis. We compared the estimates and time series plots by key demographic variables (age, sex, urbanicity) and by SUD or mental disorder history.

### Data source

Analyses were performed using secondary data from the BC Ministry of Health, namely the Medical Service Plan (physician billing), the Discharge Abstract Database (hospitalizations), and the PharmaNet (medication dispensations from community pharmacies). These databases cover all people who accessed BC's health system and are linked at the individual level by matching direct identifiers, including Personal Health Number, name and date of birth. We accessed these databases through the Provincial Health Service Authority's Platform for Analytics and Data (PANDA), which evolved from a proof-of-concept cloud platform established at the beginning of the pandemic.^13^

### Exposure

In the interrupted time series analysis, the exposure events, or interruption points, are the start and end dates of the pandemic provincial state of emergency. We used these dates to construct two sets of event indicator variables given that the initiation and termination of the pandemic measures might affect the trend of ADHD diagnoses. These indicator variables model different types of structural changes associated with each interruption point. For both the start and end of the pandemic, we estimated two types of changes: an abrupt shift in the month after the event (change in level) and a gradual linear change per month (change in slope).

### Outcome

Diagnosed adult ADHD, the outcome, was identified from the linked databases using a search algorithm (Supplementary Table S1). A case was defined as a person who had either at least two physician billing records within a year or any hospitalization records with International Classification of Diseases (ICD) codes listed in Supplementary Table S1^14^ and was aged 17 or older at the time of diagnosis, based on the Diagnostic and Statistical Manual of Mental Disorders fifth edition (DSM-5) criteria for adult ADHD. Individuals aged 16 or younger were excluded because childhood ADHD is distinguished by different criteria^15^; though adults with persistent childhood-onset ADHD could still appear as a case (only if they had a diagnosis before Jan 1, 2008, the earliest date available on PANDA). We measured the outcome as incidence rates per month where an incident case was defined as a person having their first diagnosis since Jan 1, 2008. The denominator of the rates is the monthly count of people who enrolled in the Medical Service Plan and had not been diagnosed with ADHD.

### Subgroup variables

To describe the characteristics of people with newly diagnosed ADHD, we selected demographic factors including age, sex assigned at birth, and urbanicity of residence (classified based on population in the Local Health Area, see Supplementary Table S2). These variables were extracted from records identified as individuals’ first ADHD diagnoses.

SUD and mental disorder status were included for their link with ADHD discussed above. We identified these conditions and their subcategories using search algorithms similar to the ADHD definition (Supplementary Table S1). The subcategories include opioid, stimulant, alcohol, sedative/hypnotic, cannabis, and tobacco use disorders, as well as schizophrenia, stress-related disorders, mood disorders, personality/behavioural disorders, and other/unspecified mental disorders. A case was considered to have a history of these conditions if relevant diagnoses were found within five years before their first ADHD diagnosis. To ensure our data covered this five-year window for all cases, we reported results only from Jan 1, 2013, onward, but utilized records dated back to Jan 1, 2008, for identifying diseases.

### Statistical analysis

We cross-tabulated frequencies and proportions of ADHD cases for the selected variables by periods and plotted stratified time series of incidence rates per month. A separate interrupted time series model was fitted for each stratum of the stratified time series. Note that our time series analyses excluded some subcategories of SUD and mental disorders (stimulant, Sedative or hypnotic, cannabis, tobacco use disorders and schizophrenia) as their case counts were too small for reliable rate calculations; see Supplementary Figures S2 and S3 and Supplementary Table S3 for those included but not presented in the manuscript.

In the interrupted time series analysis, we estimated the percent changes in the level and slope of ADHD incidence at the start and end of the pandemic using autoregressive integrated moving average models (ARIMA). We first utilized logarithm transformation to stabilize the variance of the ADHD incidence time series and then selected the orders of ARIMA by inspecting correlograms of the pre-pandemic segment, with final model selection based on the Bayesian Information Criterion (BIC) to favour more parsimonious models. The two sets of event indicator variables for both the start and end of the pandemic were included simultaneously in the models to estimate level and slope changes for each interruption point. A multiplicative seasonal component was also incorporated for known seasonality in ADHD diagnosis.^16^ To ensure the fitted models were statistically adequate, we checked the residuals for independence and normality of distribution using the Kolmogorov–Smirnov and Box–Ljung tests.^17^ See the Supplementary Methods section for a demonstration of the modelling process.

We kept the missing data entries in the sex (<0.1%) and urbanicity (1.4%) variables and assigned them to an “Unknown” category; the minimal amount of unknown sex values would not meaningfully affect the conclusions, while we considered the unknowns in urbanicity (i.e., missing address) to be informative and could be related to unstable housing or out of the province status. These analyses were performed in the statistical software R 4.4.0 with the ‘forecast’ package.^18^

### Ethical approval and funding source

This analysis was part of the BC Centre for Disease Control's public health functions; therefore, institutional ethical approval and informed consent were not required. We acknowledge funding from the UBC Psychiatry Stimulus Grants Initiative. The sponsor has no involvement in study design, data collection, analysis, interpretation, report writing, or publication decisions.

## Results

Between Jan 1, 2013 and Nov 30, 2023, we identified 101,266 incident cases of adult ADHD, with the majority occurring post-pandemic (Jul 1, 2021–Nov 30, 2023). People aged 25–34 contributed the highest proportion of 34% (34,814/101,266) of cases overall. Distributions of case characteristics changed over time (Table 1). While females comprised half of all cases, they represented 60% (31,281/51,868) of post-pandemic cases. The proportion of cases with a history of SUD decreased since the pandemic. Meanwhile, cases with a history of stress-related disorders increased, and cases with a schizophrenia diagnosis decreased. See Supplementary Figure S4 for a four-way stratification by age, sex, SUD/mental disorder histories, and period.

**Table 1:** Characteristics of incident adult attention-deficit/hyperactivity disorder (ADHD) cases, by diagnosis in pre-pandemic, pandemic, or post-pandemic periods.

Characteristic	Overall N = 101,266	Pre-pandemic N = 34,681^a^	Pandemic N = 14,717^a^	Post-pandemic N = 51,868^a^
**Demographics, n (%)**				
Age at the first diagnosis				
17–24	28,416 (28.1%)	11,045 (31.8%)	4517 (30.7%)	12,854 (24.8%)
25–34	34,814 (34.4%)	10,999 (31.7%)	5202 (35.3%)	18,613 (35.9%)
35–44	22,104 (21.8%)	6697 (19.3%)	2962 (20.1%)	12,445 (24.0%)
45–54	10,399 (10.3%)	3771 (10.9%)	1322 (9.0%)	5306 (10.2%)
55+	5533 (5.5%)	2169 (6.3%)	714 (4.9%)	2650 (5.1%)
Sex				
Female	53,538 (52.9%)	14,302 (41.2%)	7955 (54.1%)	31,281 (60.3%)
Male	47,670 (47.1%)	20,378 (58.8%)	6754 (45.9%)	20,538 (39.6%)
Unknown	58 (0.1%)	1 (0.0%)	8 (0.1%)	49 (0.1%)
Rurality of residence^b^				
Metro	54,068 (53.4%)	18,806 (54.2%)	8301 (56.4%)	26,961 (52.0%)
Remote	1377 (1.4%)	423 (1.2%)	228 (1.5%)	726 (1.4%)
Rural	6807 (6.7%)	2024 (5.8%)	889 (6.0%)	3894 (7.5%)
Urban/Rural	37,630 (37.2%)	12,546 (36.2%)	5141 (34.9%)	19,943 (38.4%)
Unknown	1384 (1.4%)	882 (2.5%)	158 (1.1%)	344 (0.7%)
**Substance use or mental disorder histories, n (%)**^c^				
Any substance use disorder	9426 (9.3%)	4718 (13.6%)	1280 (8.7%)	3428 (6.6%)
Opioid use disorder	3602 (3.6%)	1958 (5.6%)	445 (3.0%)	1199 (2.3%)
Stimulant use disorder	1950 (1.9%)	1036 (3.0%)	259 (1.8%)	655 (1.3%)
Alcohol use disorder	3535 (3.5%)	1594 (4.6%)	510 (3.5%)	1431 (2.8%)
Sedative or hypnotic use disorder	216 (0.2%)	119 (0.3%)	20 (0.1%)	77 (0.1%)
Cannabis use disorder	1482 (1.5%)	726 (2.1%)	219 (1.5%)	537 (1.0%)
Tobacco use disorder	363 (0.4%)	125 (0.4%)	48 (0.3%)	190 (0.4%)
Any mental disorder	63,631 (62.8%)	21,177 (61.1%)	9179 (62.4%)	33,275 (64.2%)
Schizophrenia	2353 (2.3%)	1193 (3.4%)	340 (2.3%)	820 (1.6%)
Stress related disorders	43,345 (42.8%)	13,479 (38.9%)	6299 (42.8%)	23,567 (45.4%)
Mood disorders	36,616 (36.2%)	12,851 (37.1%)	5226 (35.5%)	18,539 (35.7%)
Personality or behavioural disorders	10,433 (10.3%)	4082 (11.8%)	1471 (10.0%)	4880 (9.4%)
Other/unspecified mental disorders	29,458 (29.1%)	9420 (27.2%)	4345 (29.5%)	15,693 (30.3%)

a Pre-pandemic: Jan 1, 2013–Mar 16, 2020; Pandemic: Mar 17, 2020–Jun 30, 2021; Post-pandemic: Jul 1, 2021–Nov 30, 2023.

b Classified based on the population in Local Health Areas (similar size as school districts) containing patients' home: Remote = 0–10,000; Rural = 10,001–40,000; Urban/rural = 40,001–190,000; Metro = 190,001+.

c Defined as having at least one diagnosis of substance use or mental disorder in five years before the first ADHD diagnosis.

**Fig. 1:**
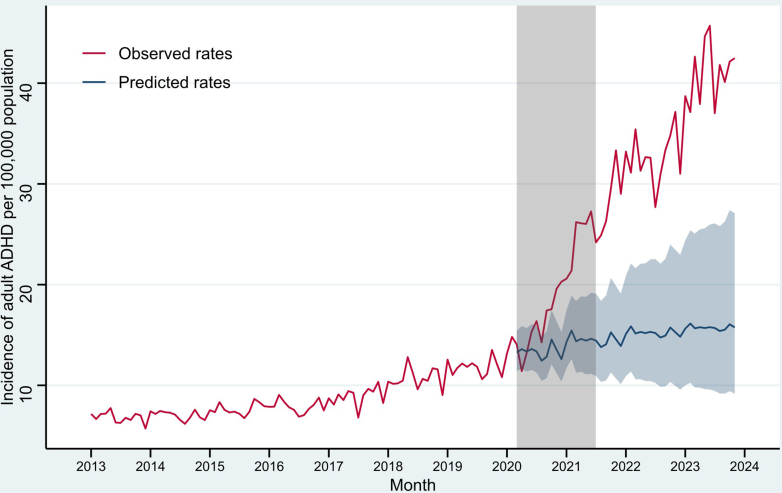
Monthly incidence of diagnosed attention-deficit/hyperactivity disorder (ADHD) among adults aged 17+ in British Columbia. The grey bar represents the pandemic period between Mar, 2020 and Jun, 2021. The predicted rates and their 95% confidence interval (the ribbon) are based on an autoregressive integrated moving average (ARIMA) model fitted to the observed rates before Mar, 2020.

The time series of diagnosed adult ADHD incidence in BC was plotted in Fig. 1 (also see Supplementary Figures S5 and S6 for an adult-adolescent trend comparison). Before the pandemic, the average rate was observed to be 8.8 cases per 100,000 population per month. This rose to 19.2 during the pandemic, a 118.2% increase, followed by a further 81.2% increase to 34.8 in the post-pandemic period.

Our interrupted time series analysis revealed these large increases at the start and end of the pandemic manifested in different patterns (Table 2). The increase during the pandemic was gradual with a 4.9% (95% confidence interval: [3.7, 6.2]) month-over-month growth (i.e., change in slope), while the latter consisted of a sudden 107.3% [68.5, 155.0] increase in the month after the end of the pandemic period (i.e., change in level) and a slower 1.5% [0.4, 2.7] growth per month thereafter. This pattern of a gradual increase during the pandemic followed by a large jump and further steady growth can be observed in most stratum-specific rates, but some strata grew faster than others.

**Table 2:** Changes in monthly rates of diagnosed adult attention-deficit/hyperactivity disorder (ADHD) between pre-pandemic, pandemic, and post-pandemic periods.

	Average monthly rate of diagnosed ADHD			Difference between pre-pandemic and pandemic			Difference between pandemic and post-pandemic		
	Pre-pandemic^a^	Pandemic^a^	Post-pandemic^a^	% change in average	Estimated % change in level^b^	Estimated % change in slope^b^	% change in average	Estimated % change in level^b^	Estimated % change in slope^b^
**All cases**	8.8	19.2	34.8	118.2%	−10.8% (−19, −1.7)	4.9% (3.7, 6.2)	81.2%	107.3% (68.5, 155)	1.5% (0.4, 2.7)
**Age**									
17–24	22.1	50.4	75.8	127.8%	−7.7% (−20.6, 7.2)	5.4% (3.7, 7.1)	50.5%	106.0% (57.7, 169.1)	0.7% (−0.7, 2.1)
25–34	15.6	37.1	67.6	138.6%	−10.0% (−20.9, 2.3)	6.1% (4.5, 7.6)	82.1%	151.4% (98.9, 217.7)	1.3% (0.2, 2.4)
35–44	10.4	22.9	47.7	119.3%	−13.3% (−27.4, 3.6)	4.8% (2.5, 7.1)	108.5%	107.0% (47, 191.4)	2.2% (0.4, 4)
45–54	5.7	11.5	24.3	101.5%	0.2% (−20.2, 26)	3.9% (1.5, 6.4)	110.3%	78.2% (26.3, 151.6)	3.1% (1.6, 4.5)
55+	1.5	2.5	4.7	62.9%	0.5% (−20.8, 27.6)	3.3% (1, 5.7)	89.4%	57.6% (14.9, 116.2)	2.9% (1.7, 4.2)
**Sex**									
Female	7.2	20.5	41.7	185.0%	−8.5% (−18.7, 2.9)	6.7% (5.1, 8.3)	103.4%	168.4% (108.8, 244.9)	1.7% (0.4, 3.1)
Male	10.4	17.8	27.7	71.3%	−9.7% (−19, 0.7)	3.3% (2, 4.6)	55.3%	58.2% (29.3, 93.7)	1.6% (0.6, 2.6)
**Any SUD history**									
Yes	41.9	55.4	79.5	32.3%	−12.0% (−26.9, 6)	3.0% (1.2, 4.9)	43.4%	48.3% (14.8, 91.7)	1.5% (0.5, 2.5)
No	7.8	18.1	33.5	130.9%	−10.5% (−19.3, −0.7)	5.1% (3.7, 6.6)	85.3%	112.9% (68.8, 168.5)	1.6% (0.3, 2.9)
**Any MD history**									
Yes	22.8	48.0	91.4	110.7%	−8.5% (−17, 0.9)	5.0% (3.8, 6.3)	90.5%	112.4% (74.3, 158.9)	1.9% (0.8, 3)
No	4.5	9.6	16.5	114.7%	−11.1% (−21.5, 0.7)	5.0% (3.5, 6.5)	71.3%	105.0% (61.8, 159.8)	1.4% (0.2, 2.7)

Notes: Pre-pandemic = Jan 1, 2013–Mar 16, 2020; Pandemic = Mar 17, 2020–Jun 30, 2021; Post-pandemic = Jul 1, 2021–Nov 30, 2023; change in level = a sudden step shift; change in slope = gradual changes per month; acronyms: ADHD, attention-deficit/hyperactivity disorder; SUD, substance use disorder; MD, mental disorder.

a Number of cases per 100,000 stratum-specific population.

b Point estimates (95% confidence intervals) from autoregressive integrated moving average models, measured in percentage of the previous month.

**Fig. 2:**
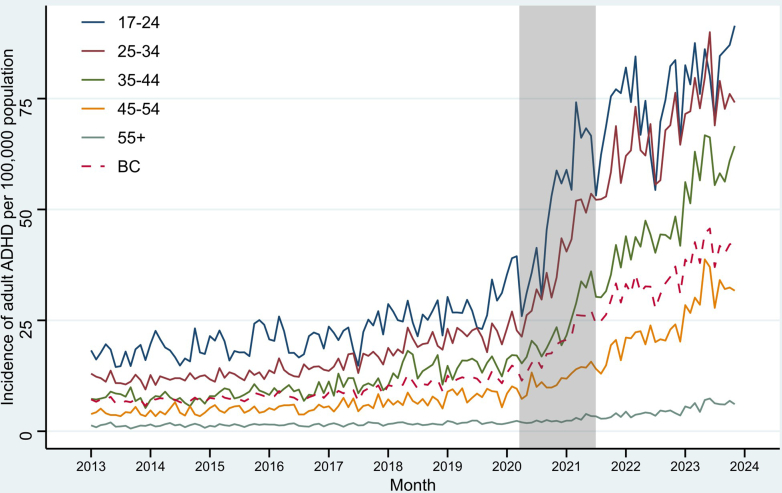
Monthly incidence of diagnosed attention-deficit/hyperactivity disorder (ADHD) among adults aged 17+ in British Columbia (BC) by age category. The grey bar represents the pandemic period between Mar, 2020 and Jun, 2021. The dashed line is the overall rate in BC.

In terms of analyses by age, all age groups had a more than tripling of incidence rates and approximately followed the pattern of the overall rates throughout the study periods, except the 17–24 group (Fig. 2). In the post-pandemic period, the growth rate of this youngest group was close to stagnant (change in slope: 0.7% [−0.7, 2.1]) and occasionally surpassed by the 25–34 group.

**Fig. 3:**
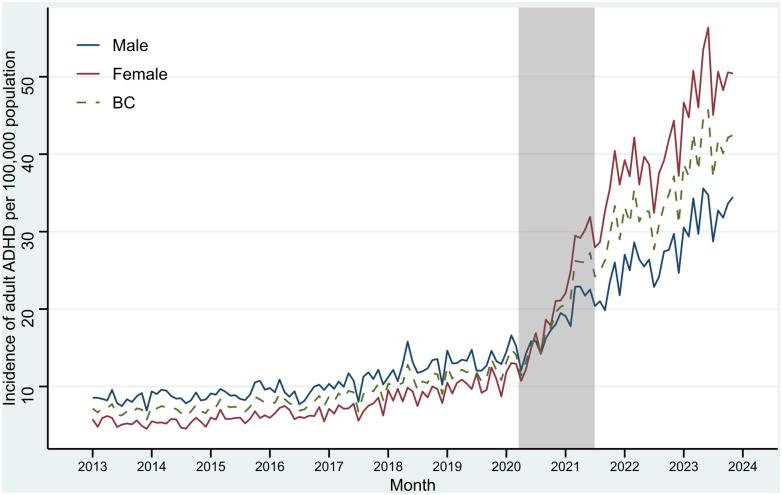
Monthly incidence of diagnosed attention-deficit/hyperactivity disorder (ADHD) among adults aged 17+ in British Columbia (BC) by sex assigned at birth. The grey bar represents the pandemic period between Mar, 2020 and Jun, 2021. The dashed line is the overall rate in BC.

When considering findings by sex, incidence rates were lower in females compared to males in the pre-pandemic period. However, rates grew by 185% in females, surpassing males since Jul 2020 (Fig. 3). During the pandemic, the estimated rate of growth for females was more than twice that (6.7% [5.1, 8.3]) of males (3.3% [2.0, 4.6]). After the pandemic, despite the almost identical growth rates by sex, females had a larger level change (168.4% [108.8, 244.9]) compared to males (58.2% [29.3, 93.7]). There were no clear differences in the level or slope changes across the study periods by urbanicity (Supplementary Figure S1).

**Fig. 4:**
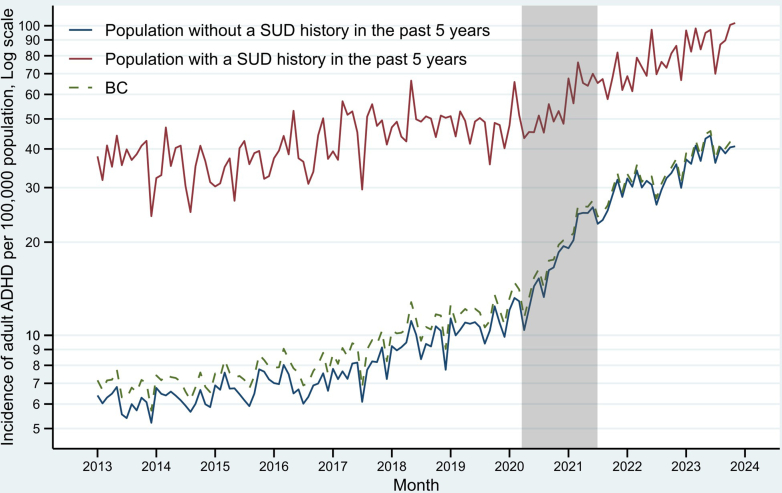
Monthly incidence of diagnosed attention-deficit/hyperactivity disorder (ADHD) among adults aged 17+ in British Columbia (BC) by substance use disorder (SUD) history status. The grey bar represents the pandemic period between Mar, 2020 and Jun, 2021. The dashed line is the overall rate in BC. Note that the y-axis is in log scale to highlight relative changes, e.g., the distance between 5 and 10 is the same as 20–40, representing a 100% increase.

Incidence rates of ADHD in those who had a history of SUD were approximately 5 times higher than people without SUD history in the pre-pandemic period (Fig. 4). This gap narrowed to about 2–3 times higher post-pandemic as the group without SUD history had larger increases in slope (5.1% [3.7, 6.6] vs. 3.0% [1.2, 4.9]) and in level (112.9% [68.8, 168.5] vs. 48.3% [14.8, 91.7]) in the pandemic and post-pandemic periods, respectively.

**Fig. 5:**
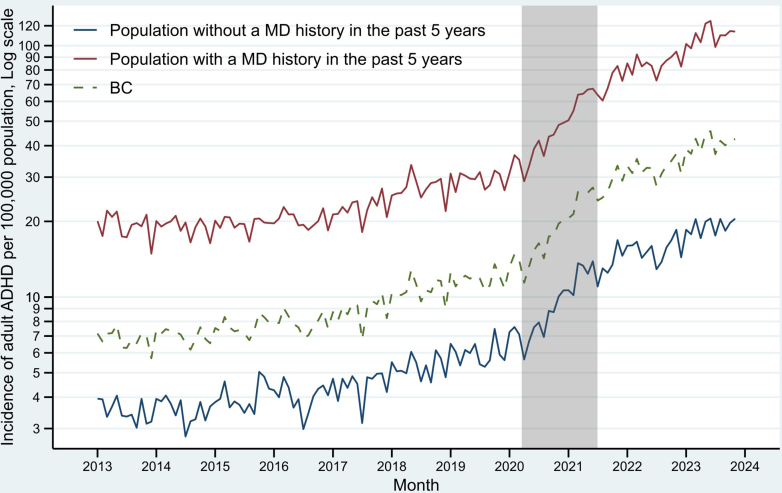
Monthly incidence of diagnosed attention-deficit/hyperactivity disorder (ADHD) among adults aged 17+ in British Columbia (BC) by mental disorder (MD) history status. The grey bar represents the pandemic period between Mar, 2020 and Jun, 2021. The dashed line is the overall rate in BC. Note that the y-axis is in log scale to highlight relative changes, e.g., the distance between 5 and 10 is the same as 20–40, representing a 100% increase.

Compared to people without a mental disorder history, incidence rates in all periods were around 5 times higher in those with a history of mental disorder. This gap between the two groups was stable as both groups followed trends similar to the overall rates in BC (Fig. 5).

## Discussion

This study shows that the rates of newly diagnosed adult ADHD in BC increased more than three-fold since the pandemic, with substantial differences in trends by sex and SUD histories. Overall, this rise aligns with the reported increase in dispensations of mental health medication in BC and diagnostic trends seen elsewhere.^19^ The relatively steady rate increase before 2020 may reflect the broadened DSM-5 diagnostic criteria released in May 2013.^20^ However, this alone cannot explain the exponential increase since the pandemic.

### Pandemic influences

The dramatic rise in diagnostic ADHD incidence since 2020 likely reflects multiple pandemic-related factors. The pandemic may have exacerbated existing subclinical ADHD symptoms, either through experiences relating to infection and post-infection recovery^21^ or through social experiences related to the pandemic response. These social experiences could include lifestyle changes and economic stressors.^22^ For example, when the pandemic began, the provincial state of emergency measures reduced activities outside and altered daily routines. As a result, people became less socially and physically active, spending more ‘forced’ time in close quarters with family.^23^ Home environments may have been stressful for parents living with school children, especially given that adults with ADHD are more likely to be parents of children with ADHD due to genetic predisposition^23^—this aligns with our observation that the 25–34 and 35–44 age groups had the highest overall increases. These stressors could be compounded by financial instability due to layoffs.^24^ When the provincial state of emergency ended, the increased incidence in the following month may reflect challenges in transitioning back to in-person contact. Returning to more structured work environments may expose previously masked ADHD symptoms during remote work, potentially increasing people's perceived severity of their symptoms.^25^

Non-etiologic factors may have also contributed by reducing barriers and stigma around seeking and receiving an ADHD diagnosis. Previous studies suggest that increased public awareness and education on ADHD in recent years have improved social acceptance and symptom recognition among both the public and practitioners.^26^ One of the drivers of public awareness of ADHD was found to be social media,^26^ which may have become even more influential as screen time increased during the pandemic. Table 3 provides a structured summary of these factors through the lens of the Health Belief Model.^27^

**Table 3:** Summary of potential pandemic impacts on adult attention-deficit/hyperactivity disorder (ADHD) incidence using the Health Belief Model.

Personal health behaviour concept	Definition	Pandemic-related sociocultural change	Direction of impact	Proposed mechanism
Perceived susceptibility	Beliefs about the chances of getting a condition	Lifestyle changes and financial instability	Increase	Stressors due to disrupted daily routines and layoffs may exacerbate subclinical-level ADHD symptoms to become problematic.
		Increased education and accessibility of information	Increase	Individuals may recognize their undiagnosed symptoms from information shared through social media or public health education.
Perceived severity	Beliefs about the seriousness and consequences of a condition	Changes in environments after the pandemic	Increase	ADHD symptoms can be more pronounced in structured and well-monitored work environments compared to remote work.
		Increased education and accessibility of information	Increase	Individuals may learn about the consequences of untreated ADHD from education or social media.
Perceived benefits	Beliefs about the effectiveness of taking action to reduce threats	Increased education and accessibility of information	Increase	Individuals can learn about the benefits of effective treatment through educational resources or social media.
Perceived barriers	Beliefs about the material and psychological costs of taking action	Increased social awareness	Decrease	Increased public awareness of ADHD and mental health may have improved social acceptance, practitioners' familiarity with diagnosis, and access to mental health services, reducing stigma and barriers around seeking and receiving a diagnosis.
Cues to action	Stimulus that prompts action	Increased education and accessibility of information	Increase	Discussions about treatment benefits in social circles or on social media may encourage individuals to recognize their symptoms and seek a diagnosis.
Self-efficacy	Confidence in one's ability to take action	Increased education and accessibility of information	Increase	Receiving education and observing the experiences of others can enhance an individual's confidence in seeking and receiving treatment.

Note: The Health Belief Model is one of the most widely used frameworks for understanding health behaviours. It posits that an individual's decision to engage in health-seeking behaviour is influenced by their perceptions of the above concepts. Definitions of these concepts are adapted from Rimer & Glanz 2020.^27^

Acronyms: ADHD, attention-deficit/hyperactivity disorder.

### Complexities in the context of SUD

An unexpected finding, given the high comorbidity of SUD and ADHD,^9^ was the slower growth of adult ADHD diagnoses among those with SUD histories. We suggest two possible explanations. First, compared to the general population, individuals with SUD may be less likely to seek care for ADHD because of personal barriers (e.g., socioeconomic disadvantages, stigma related to substance use) and structural barriers, including service access inequity and insufficient insurance coverage.^28^ Second, in clinical settings, diagnosing and treating ADHD in this population may be difficult. Regular substance use can decrease cognitive capacity, which can mask (or be mistaken for) concurrent ADHD symptoms.^29^

Even when both conditions are deemed present, ADHD may not be treated with SUD concurrently. Severe SUD often takes treatment priority due to immediate risks such as overdose.^30^ Furthermore, prescribing stimulants for ADHD to this population is complicated by factors such as drug interactions, tolerance and risk of priming when doses are too low.^29^ These complexities may delay or reduce focus on ADHD diagnosis and treatment. Untreated ADHD could drive this population to seek stimulants from unregulated sources where drug poisoning risk is high.

### Sex differences

We found rates among females increased substantially and overtook rates among males during the pandemic. This may be described as a “catching-up effect”. Historically, the diagnostic criteria for ADHD emphasized hyperactivity which is more commonly observed among males. Over time, increasing attention has been paid to the underdiagnosed inattentive subtypes that manifest more often in females.^20^ This shift toward recognizing sex differences in symptoms has likely contributed to the observed rise in diagnosis among females. In addition, adult females often carried heavier family responsibilities during the pandemic, which may have also contributed to higher rates among females.

### Limitations

This study has two key limitations. First, using diagnostic codes for disease definitions captured only people who accessed healthcare and received formal diagnoses. Consequently, our analyses likely underestimate true incidence rates—especially during the pandemic period when access to healthcare for non-urgent cases was limited, and misclassification bias may exist in the SUD/mental disorder strata as those less likely to seek healthcare and receive ADHD diagnoses are also more likely to be misclassified. The second limitation, however, might bias our estimates upward. We defined an incident case as the first diagnosis within our data time frame. Some cases we identified as new may have been previously diagnosed, indicating we may have overestimated incidence by mixing with prevalent cases. Moreover, the use of health administrative data also limited the availability of contextual information. For instance, data on whether a case was diagnosed at a virtual clinic only became available after October 2021, preventing a pre- and post-pandemic comparison (see Supplementary Table S4 for descriptives of the available data). Information on ethnicity is also absent in BC's administrative data. Ethnicity is known to be associated with ADHD diagnosis rates. While its absence does not affect the presented findings, it represents a notable gap in our list of selected subgroup variables, leaving important sociocultural factors uncovered.

### Conclusion

Given the connection between ADHD and subsequent substance use established elsewhere,^31^ the rise in adult ADHD revealed in this study may indicate that more people could be at risk of developing SUD in the years ahead, representing a critical concern amid BC's drug poisoning crisis. This situation underscores the need for bidirectional integration of ADHD and SUD services. To foresee potential impacts, future research could examine the association between ADHD and drug poisoning and how psychostimulant medications and SUD mediate this association. Future analyses should also consider temporal differences in ADHD-related outcomes by not only age but also birth cohort/decade to account for the societal evolution in mental health awareness in recent years and decades.

## Contributors

Conception—HP, KH.

Design—HP, KH.

Data acquisition—KH and HP had full access to all the data in the study.

Data analysis—KH, HP.

Interpretation—KH, HP, AC, CS, RD, SA, BZ.

Drafting manuscript—KH, HP.

Critical revision—TN, GM, AC, CS, SB, HS, JH, SA, RD, BZ, KM.

Decision to submit—HP.

Approval of the submitted version—KH, RD, SA, BZ, SB, KM, TN, CS, HS, JH, GM, AC, HP.

## Data sharing statement

The data used in this paper come from the Platform for Analytics and Data hosted by the Provincial Health Services Authority of British Columbia. Individuals interested in accessing the data should contact Dr. Heather Palis (heather.palis@bccdc.ca). Access to data provided by the Data Stewards is subject to approval but can be requested for research projects through the Data Stewards or their designated service providers. The following data sets were used in this study: Medical Service Plan, Discharge Abstract Database, PharmaNet, and Client Roster.

## Declaration of interests

Dr. Alexis Crabtree discloses the being paid on salary by PHSA, a government-funded health provider organization for her role as a public health physician, which includes epidemiology and surveillance of drug-related harms. PHSA does not direct the outputs of scientific work, including work on this manuscript.
