# Supplementary material for: Trends of incident adult Attention-deficit/hyperactivity disorder diagnoses before, during and after the pandemic provincial state of emergency in British Columbia (2013–2023): a population-based study
Source: Lancet Reg Health Am. 2025 Sep 16;51:101223. doi: 10.1016/j.lana.2025.101223 (PMC12630555; doi:10.1016/j.lana.2025.101223)
Supplement: Supplementary Figures and Tables [file mmc1.docx]

**Supplement – Trends of incident adult attention-deficit/hyperactivity disorder diagnoses before, during and after the pandemic provincial state of emergency in British Columbia (2013-2023): a population-based study**

Table of Contents

[A. Case and variable definitions 2](#_Toc204087191)

[Supplementary Table 1: Attention-deficit/hyperactivity disorder (ADHD), substance use and mental disorder case definitions 2](#_Toc204087192)

[Supplementary Table 2: Definitions for demographic variables 4](#_Toc204087193)

[B. Supplementary results 5](#_Toc204087194)

[Supplementary Figure 1: Monthly incidence of diagnosed attention-deficit/hyperactivity disorder (ADHD) among adults aged 17+ in British Columbia (BC) by urbanicity of patient’s residence 5](#_Toc204087195)

[Supplementary Figure 2: Monthly incidence of diagnosed attention-deficit/hyperactivity disorder (ADHD) among adults aged 17+ in British Columbia (BC) by subcategories of substance use disorder history 6](#_Toc204087196)

[Supplementary Figure 3: Monthly incidence of diagnosed attention-deficit/hyperactivity disorder (ADHD) among adults aged 17+ in British Columbia (BC) by subcategories of mental disorder history 7](#_Toc204087197)

[Supplementary Table 3: Changes in monthly rates of diagnosed adult attention-deficit/hyperactivity disorder (ADHD) between pre-pandemic, pandemic, and post-pandemic periods by variables not shown in the main manuscript 8](#_Toc204087198)

[C. Supplementary analyses 9](#_Toc204087199)

[Supplementary Figure 4: Four-way stratification of incident attention-deficit/hyperactivity disorder (ADHD) cases by age, sex, substance use (SUD) and mental disorder (MD) histories, and period. 9](#_Toc204087200)

[Supplementary Figure 5: Monthly incidence of diagnosed attention-deficit/hyperactivity disorder (ADHD) among adults aged 17+ and adolescents aged under 17 in British Columbia. 10](#_Toc204087201)

[Supplementary Figure 6: Monthly incidence of diagnosed attention-deficit/hyperactivity disorder (ADHD) by sex assigned at birth among adults aged 17+ and adolescents aged under 17 in British Columbia. 11](#_Toc204087202)

[Supplementary Table 4a: Type of facilities where the adult attention-deficit/hyperactivity disorder (ADHD) cases were diagnosed. 12](#_Toc204087203)

[Supplementary Table 4b: New service location codes replacing “Practitioner's office - in community” since Oct 1, 2021 13](#_Toc204087204)

[D. Supplementary Methods 14](#_Toc204087205)

[E. Reference 25](#_Toc204087206)

# Case and variable definitions

## Supplementary Table 1: Attention-deficit/hyperactivity disorder (ADHD), substance use and mental disorder case definitions

| **Condition** | **Search algorithm^1^** | **Full List of relevant ICD9/10 Codes and/or Medication**  **DIN PINs** |
| --- | --- | --- |
| **ADHD** | One or more hospitalizations with a relevant diagnostic code, OR Two or more physician visits with a relevant diagnostic code within one year. | ICD9: 314  ICD10: F90 |
| **Substance use disorder**(1) |  |  |
| Any substance use disorder | One or more hospitalizations with a relevant diagnostic code, OR Two or more physician visits with a relevant diagnostic code within one year. | ICD9: 291, 292, 303, 304, 305  ICD10: F10 - F19 |
| Opioid use disorder(2) | One or more hospitalizations with a relevant diagnostic code, OR Two or more physician visits with a relevant diagnostic or fee item codes within one year, OR One or more medication dispensations with DIN PINs related to opioid agonist treatment | ICD9: 3040, 3047, 3055  ICD10: F11  Fee item: 39, 15039, 13013, 13014, 36521  DIN PIN: 999792, 999793, 66999990, 66999991, 66999992, 66999993, 66999997, 66999998, 66999999, 67000000, 67000001, 67000002, 67000003, 67000004, 67000005, 67000006, 67000007, 67000008, 67000009,67000010, 67000011, 67000012, 67000013, 67000014, 67000015, 67000016, 67000017, 67000018, 67000019, 67000020, 22123374, 2295695, 2295709, 2408090, 2408104, 2424851, 2424878, 2453908, 2453916, 2468085, 22123346, 22123347, 22123348, 22123349, 2468093, 2502313, 2502321, 2502348, 2502356, 2517175, 2517183, 2483084, 2483092, 2474921, 9858127, 9858128, 2242963, 2242964, 2146126, 22123340, 2469413, 22123357, 66123367, 66128316, 66128328, 66128314, 66128329, 66128330, 66128331, 66128315, 655619, 781460, 781479, 655627, 2524996, 2525003 |
| Stimulant use disorder | One or more hospitalizations with a relevant diagnostic code, OR Two or more physician visits with a relevant diagnostic code within one year. | ICD9: 3042, 3044, 3056, 3057  ICD10: F14, F15 |
| Alcohol use disorder | One or more hospitalizations with a relevant diagnostic code, OR Two or more physician visits with a relevant diagnostic code within one year. | ICD9: 291, 303, 3050  ICD10: F10 |
| Sedative or hypnotic use disorder | One or more hospitalizations with a relevant diagnostic code, OR Two or more physician visits with a relevant diagnostic code within one year. | ICD9: 3041, 3054  ICD10: F13 |
| Cannabis use disorder | One or more hospitalizations with a relevant diagnostic code, OR Two or more physician visits with a relevant diagnostic code within one year. | ICD9: 3043, 3052  ICD10: F12, T407 |
| Tobacco use disorder | One or more hospitalizations with a relevant diagnostic code, OR Two or more physician visits with a relevant diagnostic code within one year. | ICD9: 3051  ICD10: F17 |
| **Mental disorders**(3) |  |  |
| Any mental disorder | One or more diagnoses of any of the conditions listed below | All of the below |
| Schizophrenia and delusional disorders | One or more hospitalizations with a relevant diagnostic code, OR Two or more physician visits with a relevant diagnostic code within one year. | ICD9: 295, 297, 298  ICD10: F20 - F29 |
| Mood disorder | One or more hospitalizations with a relevant diagnostic code, OR Two or more physician visits with a relevant diagnostic code within one year. | ICD9: 296, 3004, 311  ICD10: F30 - F39 |
| Stress-related disorders | One or more hospitalizations with a relevant diagnostic code, OR Two or more physician visits with a relevant diagnostic code within one year. | ICD9: 300 minus 3004, 306, 3078, 308, 309  ICD10: F40 - F48 |
| Personality or behavioural disorders (excluded ADHD) | One or more hospitalizations with a relevant diagnostic code, OR Two or more physician visits with a relevant diagnostic code within one year. | ICD9: 316, 301, 302, 307 minus 3078, 312, 313  ICD10:  F50 – F59 (behavioral syndromes associated with physiological disturbances and physical factors),  F60 – F69 (disorders of adult personality and behavior),  F91 – F98 (behavioral and emotional disorders with onset usually occurring in childhood and adolescence) |
| Other or unspecified mental disorders | One or more hospitalizations with a relevant diagnostic code, OR Two or more physician visits with a relevant diagnostic code within one year. | ICD9: 290, 293, 294, 310, 317, 318, 319, 299, 315, 50B  ICD10:  F00 – F09 (mental disorders due to known physiological conditions),  F70 – F79 (intellectual disabilities),  F80 – F89 (pervasive and specific developmental disorders),  F99 (unspecified mental disorder) |

^1^The same search algorithm with different relevant diagnostic codes was applied to multiple conditions. See the “Full List of relevant ICD9/10 Codes and/or Medication DIN PINs” column for the codes corresponding to each condition.

## Supplementary Table 2: Definitions for demographic variables

| **Variable** | **Definition** |
| --- | --- |
| **Sex** | Sex assigned at birth extracted from physician billing or hospitalization records that were identified as the person’s first ADHD diagnosis since Jan 1^st^, 2008.  The sex variable is a binary indicator and does not reflect gender identity. In 2018, BC introduced a third sex variable “X” option to birth certificates, which is not yet universally implemented and not currently available in our datasets.  Data on ethnicity are not available for this study. |
| **Urbanicity of residence**(4) | Derived categorical variable based on individual’s home postal code on physician billing or hospitalization records that were identified as the person’s first ADHD diagnosis since Jan 1^st^, 2008. The classification is based on the population in Local Health Areas (similar size as school districts) containing patients' homes:  Remote = 0 - 10,000;  Rural = 10,001 - 40,000;  Urban/rural = 40,001 - 190,000;  Metro = 190,001+;  Unknown = missing home address or out of province. |

# Supplementary results

## Supplementary Figure 1: Monthly incidence of diagnosed attention-deficit/hyperactivity disorder (ADHD) among adults aged 17+ in British Columbia (BC) by urbanicity of patient’s residence. The grey bar represents the pandemic period between Mar, 2020 and Jun, 2021. The dashed line is the overall rate in BC.


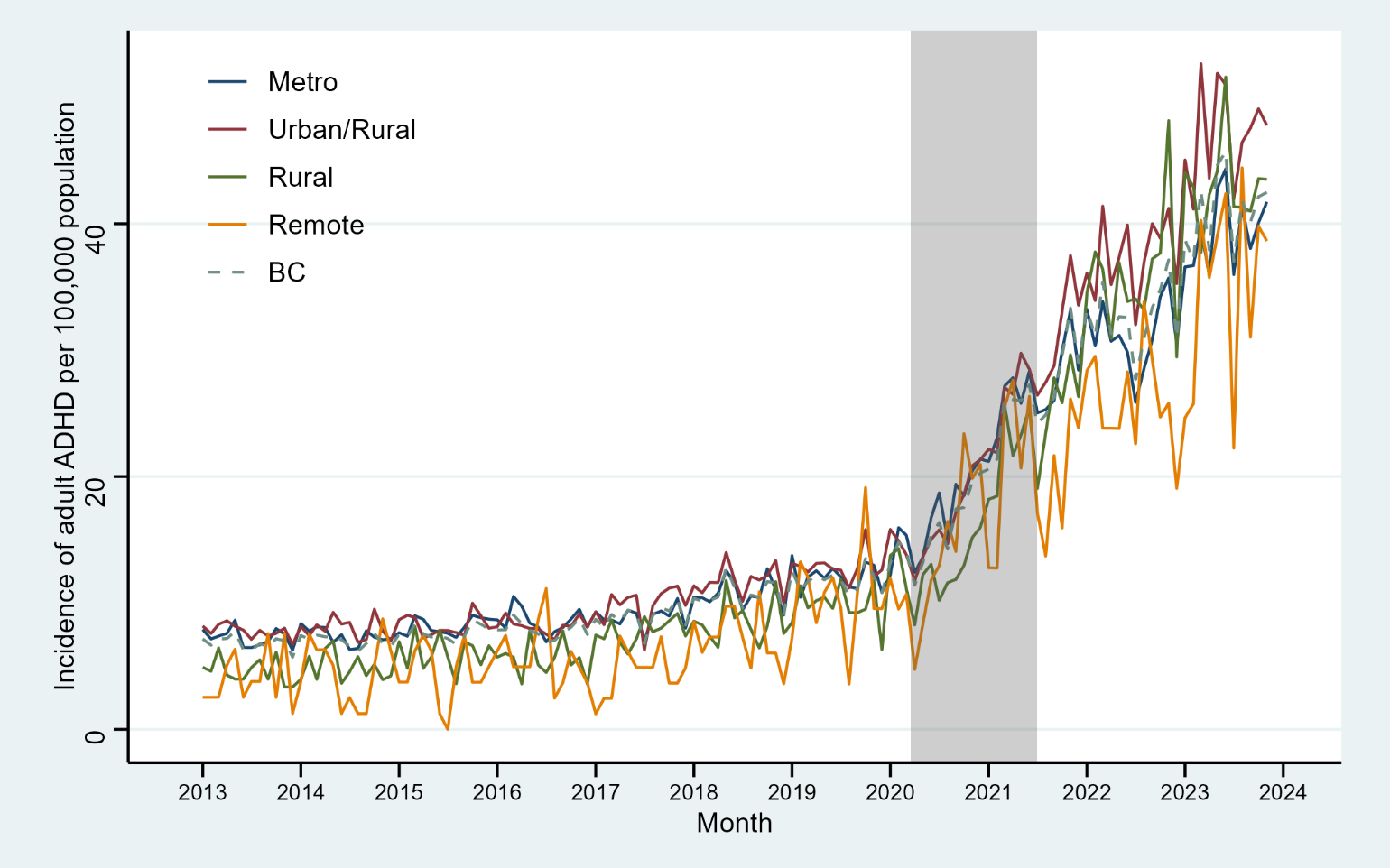


Note: The trends do not differ by urbanicity of patient’s residence.

## Supplementary Figure 2: Monthly incidence of diagnosed attention-deficit/hyperactivity disorder (ADHD) among adults aged 17+ in British Columbia (BC) by subcategories of substance use disorder history. The grey bar represents the pandemic period between Mar, 2020 and Jun, 2021. Note that y-axis is in log scale to highlight relative changes, e.g., the distance between 5 to 10 is the same as 20 to 40, representing a 100% increase.


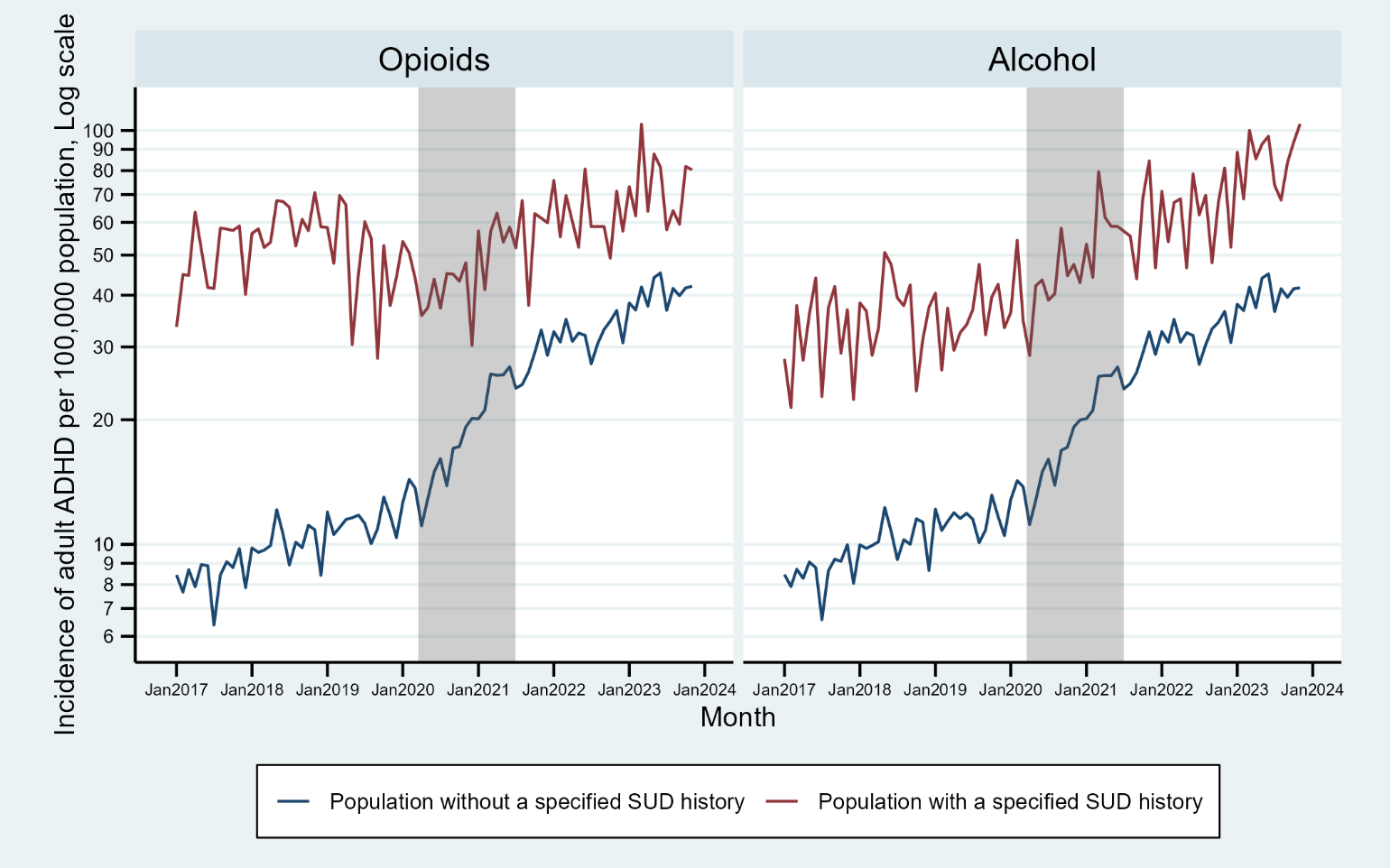


Note: These patterns are generally similar to Figure 4 in the main manuscript.

## Supplementary Figure 3: Monthly incidence of diagnosed attention-deficit/hyperactivity disorder (ADHD) among adults aged 17+ in British Columbia (BC) by subcategories of mental disorder history. The grey bar represents the pandemic period between Mar, 2020 and Jun, 2021. Note that y-axis is in log scale to highlight relative changes, e.g., the distance between 5 to 10 is the same as 20 to 40, representing a 100% increase.


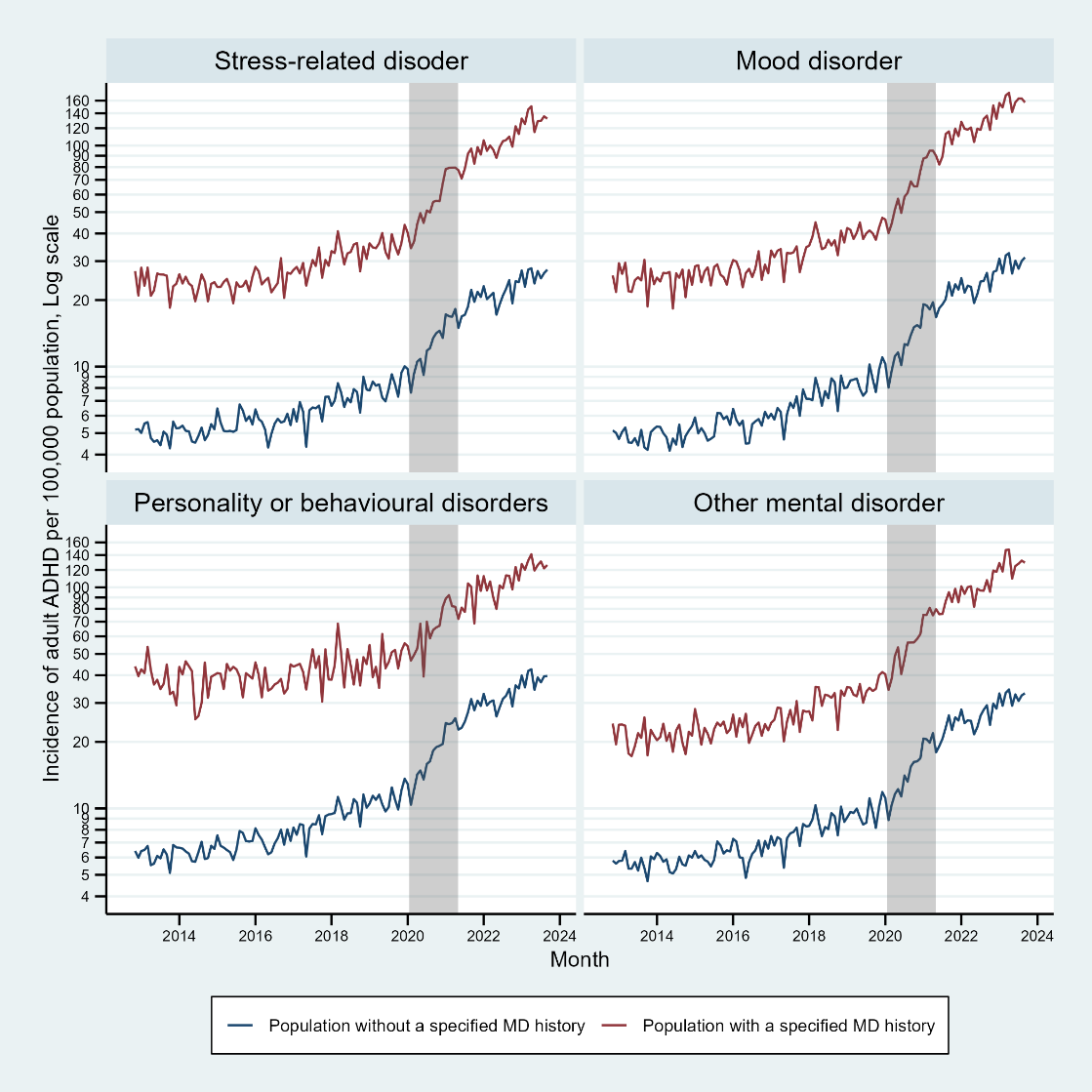


Note: These patterns are generally similar to Figure 5 in the main manuscript.

## Supplementary Table 3: Changes in monthly rates of diagnosed adult attention-deficit/hyperactivity disorder (ADHD) between pre-pandemic, pandemic, and post-pandemic periods by variables not shown in the main manuscript

|  | **Average monthly rate of diagnosed ADHD** | | | **Difference between pre-pandemic and pandemic** | | | **Difference between pandemic and post-pandemic** | | |
| --- | --- | --- | --- | --- | --- | --- | --- | --- | --- |
|  | Pre-pandemic*^1^* | Pandemic*^1^* | Post-pandemic*^1^* | % change in average | Estimated % change in level*^2^* | Estimated % change in slope*^2^* | % change in average | Estimated % change in level*^2^* | Estimated % change in slope*^2^* |
| **All cases** | | | | | | | | | |
|  | 8.8 | 19.2 | 34.8 | 118.2% | -10.8% (-19, -1.7) | 4.9% (3.7, 6.2) | 81.2% | 107.3% (68.5, 155) | 1.5% (0.4, 2.7) |
| **Urbanicity of residence** | | | | | | | | | |
| Metro | 9.1 | 20.3 | 33.7 | 122.5% | -10.0% (-21.8, 3.6) | 4.4% (1.7, 7.2) | 65.7% | 76.2% (13.5, 173.4) | 1.4% (-1.2, 4) |
| Urban/Rural | 9.7 | 19.9 | 39.8 | 106.1% | -16.8% (-26.1, -6.3) | 5.5% (4.1, 7) | 99.7% | 135.2% (87.1, 195.5) | 1.8% (0.7, 3) |
| Rural | 6.9 | 16.0 | 36.4 | 130.8% | -17.7% (-37.6, 8.6) | 6.9% (3.9, 9.9) | 128.2% | 175.5% (80, 321.6) | 2.3% (0.6, 4.1) |
| **Opioid use disorder** | | | | | | | | | |
| Yes | 53.0 | 46.2 | 65.6 | -12.7% | -30.6% (-43.7, -14.5) | 2.8% (0.7, 4.9) | 41.9% | 52.7% (19.3, 95.4) | 1.1% (0.2, 1.9) |
| No | 10.1 | 18.8 | 34.4 | 86.2% | -14.7% (-24.2, -4.1) | 5.0% (2.6, 7.4) | 82.6% | 97.5% (32.9, 193.4) | 1.7% (-0.2, 3.6) |
| **Alcohol use disorder** | | | | | | | | | |
| Yes | 35.6 | 48.6 | 71.6 | 36.2% | -2.9% (-18.9, 16.1) | 4.1% (2.3, 6) | 47.5% | 55.9% (26.7, 91.7) | 1.9% (1.2, 2.6) |
| No | 10.3 | 18.8 | 34.3 | 82.5% | -15.7% (-24.7, -5.5) | 3.8% (0.9, 6.9) | 82.5% | 65.6% (2.6, 167.4) | 0.5% (-3.6, 4.7) |
| **Stress-related disorder** | | | | | | | | | |
| Yes | 27.6 | 56.4 | 107.7 | 104.3% | -10.9% (-20.6, 0) | 5.6% (4.3, 6.9) | 90.9% | 123.6% (83.1, 173.2) | 2.0% (1, 3) |
| No | 6.1 | 12.9 | 22.3 | 109.4% | -6.6% (-16.2, 4.2) | 4.6% (3.2, 6) | 72.9% | 97.6% (56.9, 148.7) | 1.4% (0.1, 2.7) |
| **Mood disorder** | | | | | | | | | |
| Yes | 30.5 | 65.7 | 129.3 | 115.6% | -8.3% (-18.1, 2.7) | 5.4% (4.2, 6.7) | 96.7% | 125.6% (85.8, 174) | 2.0% (1.1, 3) |
| No | 6.2 | 13.8 | 24.7 | 123.0% | -10.6% (-20.4, 0.4) | 4.9% (3.3, 6.4) | 79.0% | 104.5% (60.1, 161.2) | 1.6% (0.2, 2.9) |
| **Personality or behavioural disorders** | | | | | | | | | |
| Yes | 41.6 | 66.5 | 107.0 | 59.7% | -3.1% (-19.7, 17) | 4.3% (2.5, 6.2) | 60.8% | 78.0% (40.9, 124.7) | 1.8% (0.9, 2.7) |
| No | 8.0 | 17.8 | 32.5 | 123.4% | -10.4% (-18.8, -1.1) | 5.1% (3.8, 6.4) | 82.8% | 112.8% (72.9, 162) | 1.5% (0.4, 2.7) |
| **Other mental disorders** | | | | | | | | | |
| Yes | 25.7 | 56.2 | 105.5 | 118.3% | -5.1% (-17.5, 9.2) | 5.0% (3.4, 6.5) | 87.8% | 115.8% (71, 172.3) | 1.9% (0.8, 3) |
| No | 7.1 | 15.0 | 26.9 | 112.7% | -10.9% (-19.8, -1.1) | 5.2% (3.8, 6.5) | 79.2% | 109.5% (68.4, 160.7) | 1.6% (0.4, 2.8) |
| *^1^*Number of cases per 100,000 stratum-specific population | | | | | | | | | |
| *^2^*Point estimates (95% confidence intervals) from autoregressive integrated moving average models, measured in percentage of the previous month. | | | | | | | | | |
| Notes: Pre-pandemic = Jan 1, 2013 - Mar 16, 2020; Pandemic = Mar 17, 2020 - Jun 30, 2021; Post-pandemic = Jul 1, 2021 - Nov 30, 2023; change in level = a sudden step shift; change in slope = gradual changes per month. The patterns in this table are generally similar to Table 2 in the main manuscript | | | | | | | | | |

# Supplementary analyses

## Supplementary Figure 4: Four-way stratification of incident attention-deficit/hyperactivity disorder (ADHD) cases by age, sex, substance use (SUD) and mental disorder (MD) histories, and period.

**
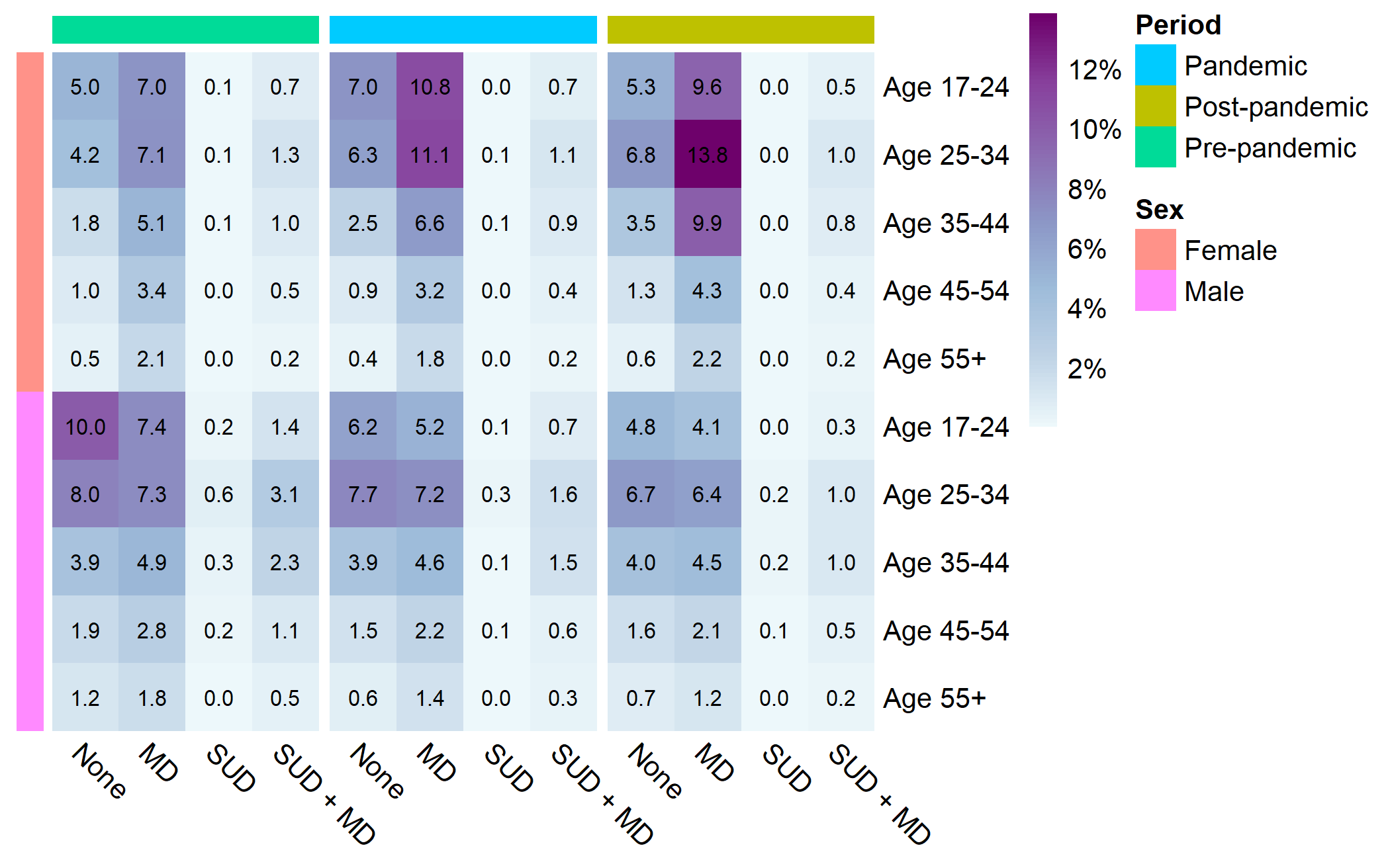
**

Note: The number in cells is the percentage of cases in a period, i.e., all cells in a period add up to 100. This quilt plot reveals that the profile of adult ADHD incident cases changed over time. Before the pandemic, nearly one-third of the cases were males under age of 35 with MD only or no SUD/MD histories. The weight of this group declined since the pandemic. In the post-pandemic period, the group with the highest weight was females under age of 45 with previous MD diagnoses.

## Supplementary Figure 5: Monthly incidence of diagnosed attention-deficit/hyperactivity disorder (ADHD) among adults aged 17+ and adolescents aged under 17 in British Columbia. The grey bar represents the pandemic period between Mar, 2020 and Jun, 2021. Note that the y-axis is in log scale to highlight relative changes, e.g., the distance between 5 to 10 is the same as 20 to 40, representing a 100% increase.


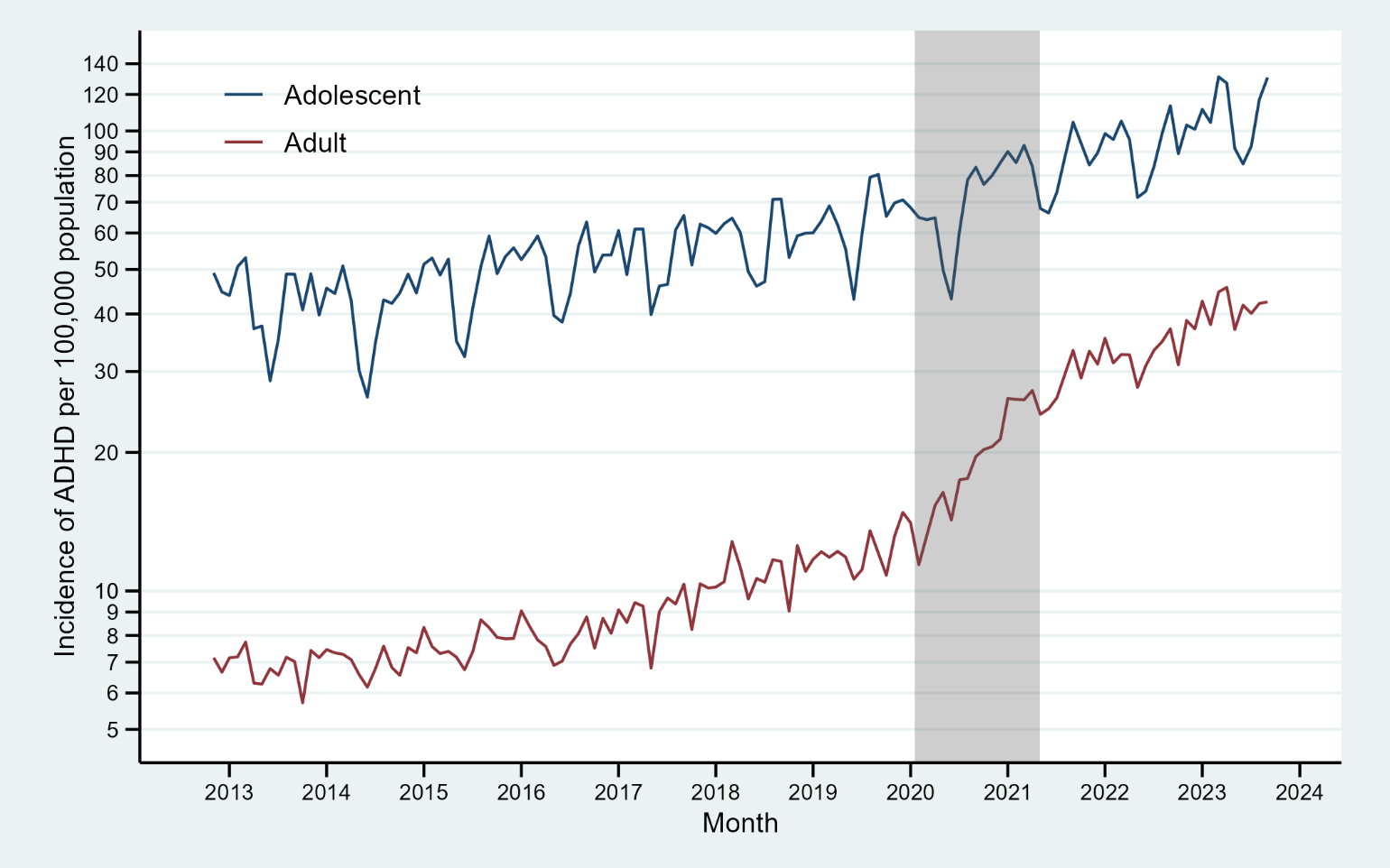


Note: The rates grew faster in adults than in adolescents since the pandemic.

## Supplementary Figure 6: Monthly incidence of diagnosed attention-deficit/hyperactivity disorder (ADHD) by sex assigned at birth among adults aged 17+ and adolescents aged under 17 in British Columbia. The grey bar represents the pandemic period between Mar, 2020 and Jun, 2021. Note that the y-axis is in log scale to highlight relative changes, e.g., the distance between 5 to 10 is the same as 20 to 40, representing a 100% increase.


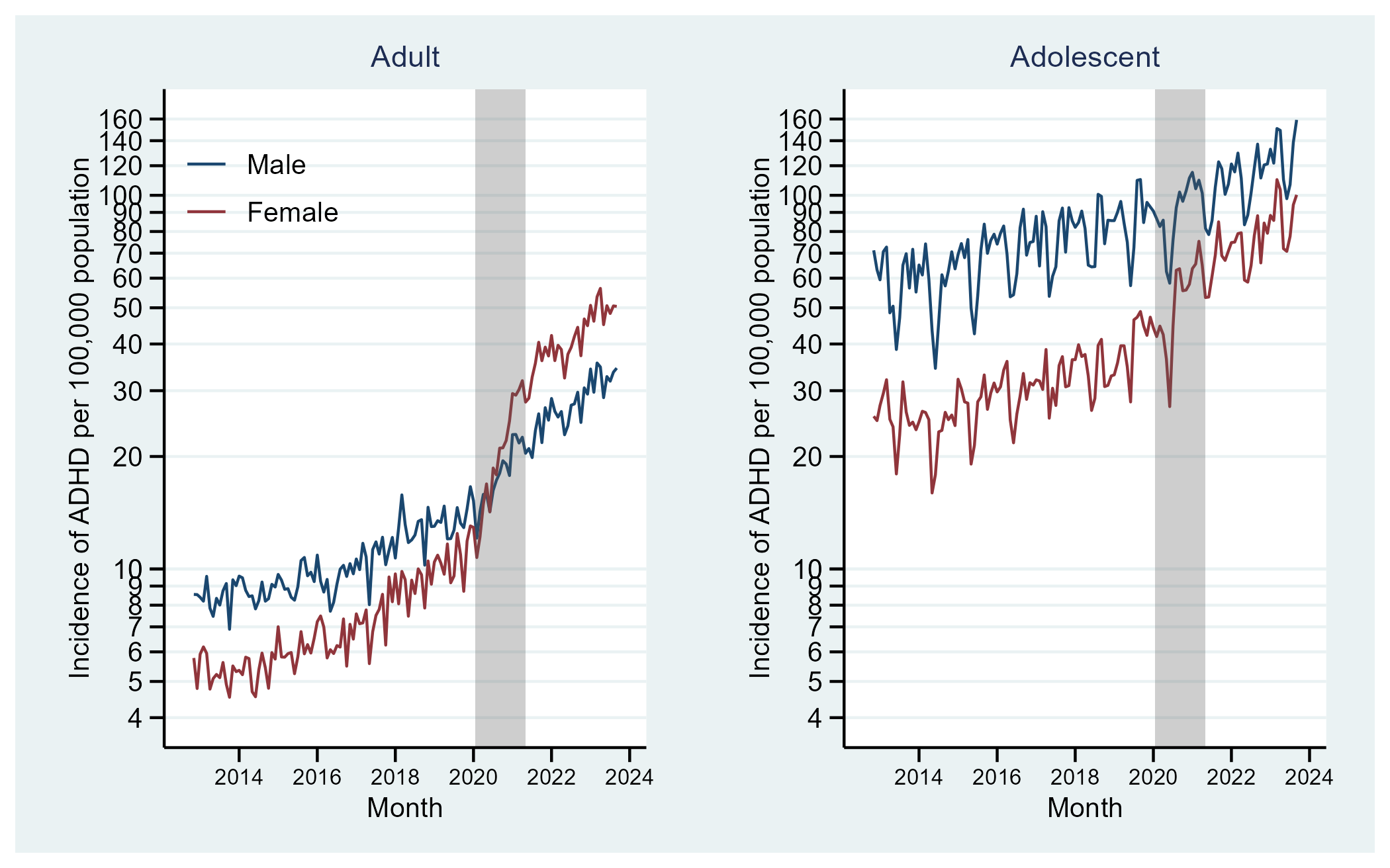


Note: The rates grew faster in females than in males among both adults and adolescents. However, females’ rates did not overtake males among adolescents.

## Supplementary Table 4a: Type of facilities where the adult attention-deficit/hyperactivity disorder (ADHD) cases were diagnosed.

| **Characteristic** | **Pandemic**, N = 14,717*^1^* | **Post-pandemic**, N = 51,868*^1^* | **Pre-pandemic**, N = 34,681*^1^* |
| --- | --- | --- | --- |
| **Service location code** |  |  |  |
| Practitioner's office - in community | 13,391 (93%) | 48,407 (95%) | 30,429 (91%) |
| Residential care/assisted living residence | 52 (0.4%) | 57 (0.1%) | 160 (0.5%) |
| Diagnostic facility | 1 (<0.1%) | 2 (<0.1%) | 11 (<0.1%) |
| Hospital - emergency room | 13 (<0.1%) | 48 (<0.1%) | 37 (0.1%) |
| Private medical/surgical facility | 4 (<0.1%) | 28 (<0.1%) | 21 (<0.1%) |
| Hospital - day care (surgery) | 1 (<0.1%) | 1 (<0.1%) | 0 (0%) |
| Hospital - inpatient | 103 (0.7%) | 175 (0.3%) | 362 (1.1%) |
| Mental health centre | 401 (2.8%) | 958 (1.9%) | 725 (2.2%) |
| Hospital - outpatient | 209 (1.4%) | 630 (1.2%) | 801 (2.4%) |
| Patient's private home | 8 (<0.1%) | 8 (<0.1%) | 17 (<0.1%) |
| Practitioner's office - in publicly administered facility | 157 (1.1%) | 770 (1.5%) | 687 (2.1%) |
| Others (i.e. accident site or in an ambulance etc.) | 81 (0.6%) | 86 (0.2%) | 123 (0.4%) |
| Unknown | 296 | 698 | 1,308 |
| *^1^*n (%) | | | |

## Supplementary Table 4b: New service location codes replacing “Practitioner's office - in community” since Oct 1, 2021

| **Characteristic** | **OCT2021 - SEP2022**, N = 17,815*^1^* | **OCT2022 - SEP2023**, N = 22,822*^1^* |
| --- | --- | --- |
| **Service Location** |  |  |
| Community Health Centre | 632 (3.5%) | 617 (2.7%) |
| Hybrid Primary Care Practice (part-time longitudinal/walk-in clinic) | 2,781 (16%) | 2,977 (13%) |
| Longitudinal Primary Care Practice (e.g. GP family practice or PCN clinic) | 12,018 (67%) | 16,375 (72%) |
| Health Care Practitioner Office (non-physician) | 41 (0.2%) | 15 (<0.1%) |
| Specialist Physician Office | 953 (5.3%) | 1,549 (6.8%) |
| Urgent and Primary Care Centre | 140 (0.8%) | 129 (0.6%) |
| Virtual Care Clinic | 950 (5.3%) | 926 (4.0%) |
| Walk-in Clinic | 300 (1.7%) | 234 (1.0%) |
| *^1^*n (%)  Note: The location of diagnosis is based on the “service location” variable in the Medical Service Plan (MSP) database. Supplementary Table 4a shows that more than 90% of the adult ADHD cases were diagnosed in practitioner's offices in communities. Codes for the subcategories of “Practitioner's office - in community” were introduced in Oct, 2021. Since then, of the cases diagnosed in practitioner's offices in communities, only about 5% were diagnosed by virtual care clinics (Supplementary Table 4b), indicating that telemedicine was not a driver of the rise of adult ADHD since the pandemic. | | |

# Supplementary Methods

Here we illustrate the approach used to estimate the changes in level and slope of ADHD rate time series using ARIMA models. The overall incidence rate of adult ADHD in BC shown in Figure 1 was used in the following example. The ADHD case definition algorithm in Supplementary Table 1 was implemented using R package ‘healthdb’ (https://cran.r-project.org/web/packages/healthdb/index.html).

The structure of ARIMA models was determined using the pre-pandemic period. Because we conceptualize that such model represents the underlying data generating process before the pandemic, and the start and end of the pandemic are external shocks to this underlying process. Therefore, predictions for time after the start of the pandemic from this model without shocks (intervention variables shown below) represent the counterfactual scenario if the pandemic did not happen.

*Determining ARIMA structure in the pre-pandemic period*

1. Visual check of the pre-pandemic segment indicates that the mean is non-stationary. Differencing is needed.

# load required packages
pacman::p_load(tidyverse, forecast, astsa)

# convert the data frame containing monthly rates into ts object
ts_full <- ts(plot_data_bc$rate_total, frequency = 12, start = c(2013, 1))

# extract the segment of the pre-pandemic period
pre <- window(ts_full, end = c(2020,2))

plot(pre, ylab = "Incidence rate per 100,000 population", xlab = "")


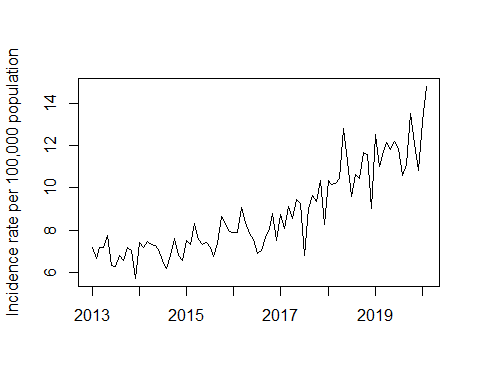


Supplementary Figure 7 Incidence rate of adult ADHD diagnoses in BC in the pre-pandemic period (January 2013 - March 2020)

1. The differenced series suggests the variance is also non-stationary. The variance is apparently larger in later years when rates were relatively high.

pre_diff <- diff(pre)
plot(pre_diff, xlab = "", ylab = "Differenced rate")


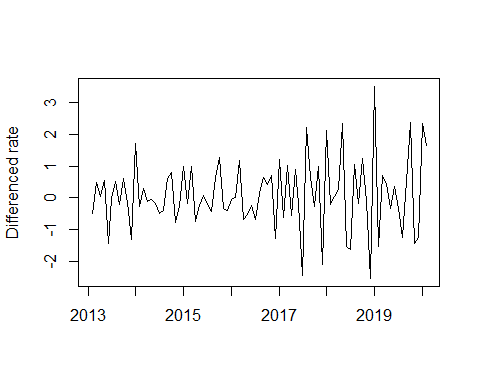


Supplementary Figure 8 First difference of adult ADHD diagnosis rates in BC in the pre-pandemic period (January 2013 - March 2020)

1. Log transformation is used to stabilize the variance

log_pre <- log(pre)
plot(diff(log_pre), ylab = "Diff(Log(rate))", xlab = "")


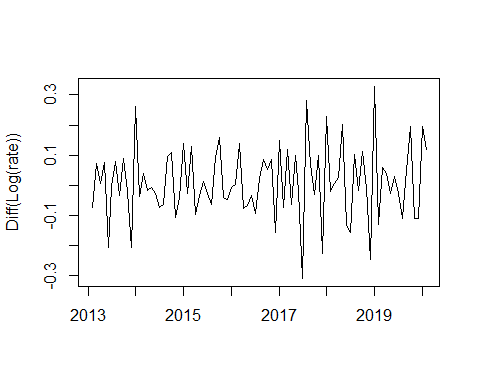


Supplementary Figure 9 First difference of the log-transformed rates of adult ADHD diagnoses in BC in the pre-pandemic period (January 2013 - March 2020)

1. By judging ACF/PACF, an ARIMA(0, 1, 1)x(0, 0, 3)_12_ model may be appropriate.

par(mfrow = c(2, 2))
Acf(diff(log_pre), lag.max = 36, main = "Diff(Log(ADHD rate))")
Pacf(diff(log_pre), main = "Diff(Log(ADHD rate))")
Acf(diff(log_pre, 12), main = "Diff-12th(Log(ADHD rate))")
Pacf(diff(log_pre, 12), main = "Diff-12th(Log(ADHD rate))")


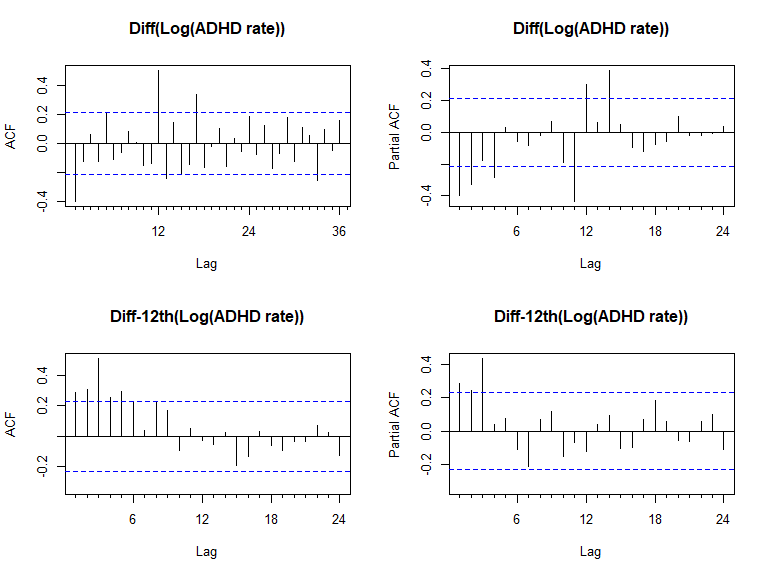


Supplementary Figure 10 Autocorrelation functions (ACF) and partial autocorrelation functions (PACF) of the time series shown in Figure S9.

par(mfrow = c(1, 1))

1. We searched for the best fitting model based on BIC (BIC tends to prefer more parsimonious models). The resulting model has an ARIMA(0, 1, 1)x(1, 0, 0)_12_ structure. We will adopt this model if it is more parsimonious than our speculation and passes the tests in the following section.

- The model search was done using the ‘auto.arima’ function in the ‘forecast’ package. Here shows the output of the ‘auto.arima’ function for the log-transformed pre-pandemic rates:

m_pre <- forecast::auto.arima(log_pre, stepwise = FALSE, ic = "bic")
m_pre

Series: log_pre
ARIMA(0,1,1)(1,0,0)[12]

Coefficients:
 ma1 sar1
 -0.7272 0.6463
s.e. 0.0702 0.0814

sigma^2 = 0.005867: log likelihood = 95.18
AIC=-184.36 AICc=-184.06 BIC=-177.03

*Fitting ITS model*

We created four intervention (dummy) variables that represent the start and end of the pandemic:

- pandemic_lv where months since Mar, 2020 have value of 1, and 0 elsewhere.
- post_pandemic_lv where months since Jul, 2021 have value of 1, and 0 elsewhere.
- pandemic_trend an incremental sequence starting from 1 between Mar, 2020 and Jun, 2021, representing a linear trend during the pandemic.
- post_pandemic_trend an incremental sequence starting from 1 since Jul, 2021, representing a linear trend after the pandemic.

These variables were included as x aggressors in an ARIMA model with the (p, d, q) structure determined from previous steps, and we interpreted their coefficients as the changes in level and trend (i.e., slope), respectively, assuming the level change happened immediately and the trend changed linearly.

Here is a summary of the model and diagnostics:

# get the index or row number for the start/end of the pandemic
cutoff1 <- which(plot_data_bc$time == "2020-03-01")
cutoff2 <- which(plot_data_bc$time == "2021-07-01")

# create the intervention variables as described above in the original data frame
model_data_1 <- plot_data_bc %>%
 select(time, rate = rate_total) %>%
 mutate(t = time(rate),
 pandemic_lv = if_else(t < cutoff1, 0, 1),
 pandemic_trend = if_else(cutoff1 <= t & t < cutoff2, t - cutoff1 + 1, 0),
 post_pandemic_lv = if_else(t < cutoff2, 0, 1),
 post_pandemic_trend = if_else(t < cutoff2, 0, t - cutoff2 + 1))

# fit model with x regressors to the full study period to get estimates for the intervention variables
y_ts <- ts(model_data_1$rate, start = c(2013,1), frequency = 12)

m1 <- sarima(log(y_ts), 0, 1, 1, 1, 0, 0, 12, xreg = data.frame(pandemic_lv = model_data_1$pandemic_lv, pandemic_trend = model_data_1$pandemic_trend, post_pandemic_lv = model_data_1$post_pandemic_lv, post_pandemic_trend = model_data_1$post_pandemic_trend), gg = TRUE, col = 4)

<><><><><><><><><><><><><><>

Coefficients:
 Estimate SE t.value p.value
ma1 -0.7583 0.0615 -12.3357 0.0000
sar1 0.5675 0.0742 7.6497 0.0000
pandemic_lv -0.1139 0.0492 -2.3130 0.0224
pandemic_trend 0.0482 0.0063 7.6554 0.0000
post_pandemic_lv 0.7289 0.1057 6.8936 0.0000
post_pandemic_trend 0.0152 0.0060 2.5512 0.0120

sigma^2 estimated as 0.006216952 on 124 degrees of freedom

AIC = -2.092768 AICc = -2.087515 BIC = -1.938363


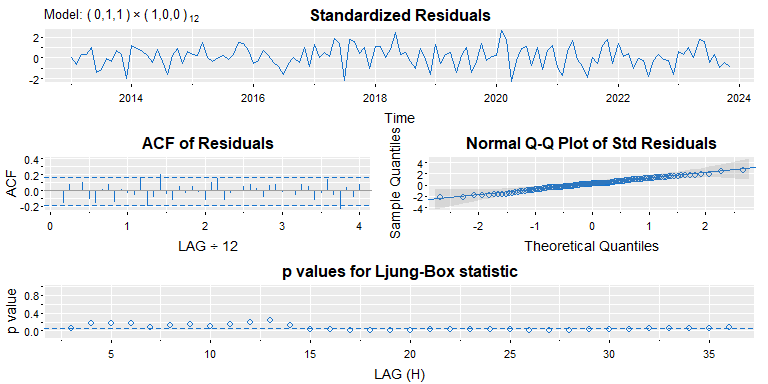


Supplementary Figure 11 Model diagnostic plots for an autoregressive moving average model fitted to the log-transformed rates of adult ADHD diagnoses in BC between January 2013 and November 2023.

The residuals seem relatively well behaved. We further confirmed normality and independence of the residuals with Kolmogorov-Smirnov test and Box-Ljung test. The following result shows both tests failed to reject the null of normality or independence.

ks.test(residuals(m1$fit), "pnorm", mean = mean(residuals(m1$fit)), sd = sd(residuals(m1$fit)))

Asymptotic one-sample Kolmogorov-Smirnov test

data: residuals(m1$fit)
D = 0.042219, p-value = 0.9737
alternative hypothesis: two-sided

Box.test(residuals(m1$fit), lag = 24, type = "Ljung")

Box-Ljung test

data: residuals(m1$fit)
X-squared = 35.65, df = 24, p-value = 0.05933

The model (in red) also fitted the original series well:

# plot the observed vs predicted (obs+residual) series
plot(log(y_ts), ylab = "Log(ADHD rate)", xlab = "")
lines(log(y_ts) + residuals(m1$fit), col = "red")


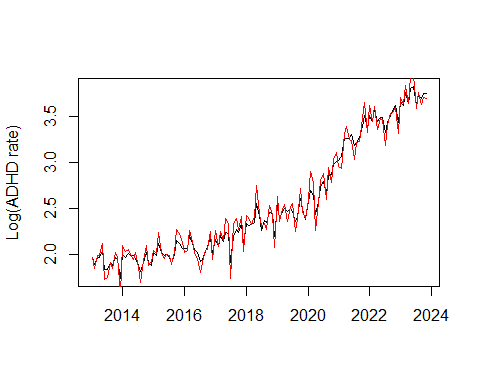


Supplementary Figure 12 Observed (black) and model-predicted value (red) of the log-transformed rates of adult ADHD diagnoses in BC in between January 2013 and November 2023.

Example interpretation of the coefficients: The test results suggest the ARIMA model is statistically adequate. For the pre-pandemic vs. pandemic comparison, the coefficient for the immediate change in level (the pandemic_lv variable) is estimated to be -0.1139 on a log scale, which is exp(-0.1139) = 0.892 on the original scale representing a 10.8% decrease. The change in slope (the pandemic_trend variable) estimate is exp(0.0482) = 1.049, a 4.9% increase per month. When the pandemic ended, the level (the post_pandemic_lv variable) increased by exp(0.7289) - 1 = 107.3% while the slope (the post_pandemic_trend variable) increased by exp(0.0152) - 1 = 1.5% per month.

# Reference

1. Gan WQ, Buxton JA, Scheuermeyer FX, Palis H, Zhao B, Desai R, et al. Risk of cardiovascular diseases in relation to substance use disorders. Drug Alcohol Depend. 2021 Dec 1;229:109132.

2. Desai R, Hu K, Xavier C, Zhao B, Palis H, Slaunwhite A, et al. New opioid use disorder diagnosis rates in British Columbia (2010-2023) [Internet]. Vancouver, BC: BC Centre for Disease Control; 2024 [cited 2024 Nov 30]. Available from: http://www.bccdc.ca/Health-Professionals-Site/Documents/KnowledgeUpdate_OUD_24July2024.pdf

3. Palis H, Hu K, Rioux W, Korchinski M, Young P, Greiner L, et al. Association of Mental Health Services Access and Reincarceration Among Adults Released From Prison in British Columbia, Canada. JAMA Netw Open. 2022 Dec 15;5(12):e2247146.

4. BC Ministry of Health. Geographic Service Areas [Internet]. 2016 [cited 2024 Aug 29]. Available from: https://www2.gov.bc.ca/assets/gov/health/about-bc-s-health-care-system/health-priorites/geographic-service-areas.docx
